# Cancer incidence among Armenians in California

**DOI:** 10.1002/cam4.7100

**Published:** 2024-03-16

**Authors:** Ani S. Movsisyan Vernon, Jeffrey S. Hoch, Laura Fejerman, Theresa H. Keegan

**Affiliations:** ^1^ Department of Public Health Sciences University of California Davis Davis California USA; ^2^ UC Davis Comprehensive Cancer Center University of California Davis Medical Center Sacramento California USA; ^3^ Center for Healthcare Policy and Research University of California Davis California USA

**Keywords:** Armenian, California, cancer, incidence, proportion

## Abstract

**Introduction:**

California is home to the largest population of Armenians in the United States. The historical categorization of Armenians as ‘White’ or ‘Some Other Race’ in population databases has likely masked cancer incidence patterns in this population. This is the first study considering cancer incidence among Armenians in California.

**Methods:**

We used the Armenian Surname List and birthplace information in the California Cancer Registry to identify Armenians with cancer diagnosed during 1988–2019. We calculated proportional incidence ratios (PIR) among Armenians compared with non‐Hispanic Whites (NHWs). As an exploratory analysis, we calculated incidence rate ratios (IRR) during 2006–2015 using Armenian population denominators from the American Community Survey (ACS). We selected PIR as our primary method given uncertainty regarding the use of ACS population estimates for rate calculations.

**Results:**

There were 27,212 cancer diagnoses among Armenians in California, 13,754 among males and 13,458 among females. Armenian males had notably higher proportions of stomach (PIR = 2.39), thyroid (PIR = 1.45), and tobacco‐related cancers including bladder (PIR = 1.53), colorectal (PIR = 1.29), and lung (PIR = 1.16) cancers. Higher proportional incidence of cancers including stomach (PIR = 3.24), thyroid (PIR = 1.47), and colorectal (PIR = 1.29) were observed among Armenian females. Exploratory IRR analyses showed higher stomach (IRR = 1.78), bladder (IRR = 1.13), and colorectal (IRR = 1.12) cancers among Armenian males and higher stomach (IRR = 2.54) cancer among Armenian females.

**Conclusion:**

We observed higher stomach, colorectal and thyroid cancer incidence among males and females, and tobacco‐related cancers among males. Further research is needed to refine Armenian population estimates and understand and address risk factors associated with specific cancers among Armenians in California.

## Abstract 2

We observed higher stomach, colorectal and thyroid cancer incidence among males and females and tobacco‐related cancers among males. Further research is needed to refine Armenian population estimates and understand and address risk factors associated with specific cancers among Armenians in California.

## 1 INTRODUCTION

California is the largest and most diverse state in the United States,^1^, ^2^ and home to the largest population of Armenians in the United States, with immigration to the state dating back to the 1870s.^3^, ^4^ Given California's racial and ethnic diversity, numerous recent research studies have focused on cancer occurrence patterns in specific ethnic groups, including Hispanic, Asian, Middle‐Eastern, and Arab populations in California.^5^, ^6^, ^7^, ^8^, ^9^ However, Armenians remain unrepresented in public health and epidemiological research due to their categorization as ‘White’ or ‘Some Other Race’ in population databases.^3^, ^10^, ^11^

Prior studies on cancer occurrence among Middle‐Eastern populations in California included the Armenian population and found proportionally higher stomach and thyroid cancers and proportionally lower lung and cervical cancers among Middle‐Eastern females compared with non‐Hispanic White (NHW) females.^9^, ^12^ In addition, proportionally higher thyroid, bladder, and stomach cancers and lower lung cancer were observed among Middle‐Eastern males compared with NHW males.^9^, ^13^ While Armenians were included in the broad Middle‐Eastern group, cancer risk factors, including tobacco‐use, are higher in Armenia compared to surrounding countries and cancer mortality rates in Armenia are notably higher than neighboring Middle‐Eastern countries, such as Iran and Iraq.^14^, ^15^ Additionally, a hospital in Los Angeles county, where the majority of Armenians in California reside, found that a majority of their patients with stomach cancer were Armenian.^16^ However, state‐level analyses of cancer incidence by cancer type and sex among the Armenian population in California and in the United States have remained unknown, and it is unclear whether cancer patterns among Armenians differ from the NHW population with which they have been historically categorized in population‐based research studies.^11^, ^17^

No prior studies, to our knowledge, have studied cancer incidence specifically among the Armenian population in California.^18^ Therefore, we utilized the recently developed Armenian Surname List (ASL)^17^ and birthplace data in the population‐based California Cancer Registry to identify Armenians with cancer. Research on this growing minority group in California can reveal the cancers disproportionately impacting Armenians.

## 2 MATERIALS AND METHODS

### 2.1 Data sources

#### 2.1.1 California Cancer Registry

The California Cancer Registry (CCR) is a statewide population‐based cancer surveillance system and has collected cancer diagnoses in California since 1988, with reporting guidelines similar to those of the Surveillance, Epidemiology, and End Results (SEER) database.^19^, ^20^ Consistently meeting data quality standards, the CCR is gold certified by the North American Association of Central Cancer Registries (NAACCR) and is one of the largest cancer registries in the world.^19^, ^21^ As mandated by NAACCR, data reported to the CCR meet standards of completeness, accuracy, timeliness, and quality control measures.^21^, ^22^ The CCR provides patient demographics, tumor characteristics including primary site, behavior, histology, morphology, and stage at diagnosis, first course of treatment, country of birth, county of diagnosis, and follow‐up for vital status for all cancers, except for non‐melanoma skin cancers.^23^ For the presented analyses, we used variables including age at diagnosis, year of diagnosis, race/ethnicity, sex, tumor site, and behavior. We additionally presented demographic variables including county of diagnosis and country of birth.

#### 2.1.2 Population estimates

The American Community Survey (ACS) is a nationwide survey provided by the US Census Bureau and was used to obtain Armenian population estimates.^24^ US Census respondents were asked “What is your ancestry or ethnic origin?”, and the ancestry variable is comprised of responses to this question.^25^, ^26^, ^27^ We used this ancestry variable to retrieve population detailed tables for the Armenian population in California, and among this population, we obtained additional demographic variables, including age, sex, place of birth, and county of residence.^18^, ^24^, ^28^ Armenian population estimates in California were retrieved from the ACS 2010 and 2015 5‐year selected population detailed tables and included sex, age, and county variables, with data available for 19 of the 58 counties in California.^18^, ^24^ Population estimates for the NHW population were obtained from the CCR, provided by the California Department of Finance and the Centers for Disease Control and Prevention National Center for Health Statistics branch.^29^, ^30^

### 2.2 Study population

To identify Armenian cancer diagnoses, we used Match*Pro probabilistic linkage software to link the ASL with the CCR December 2021 incidence research file extract that includes primary cancers considered complete for diagnosis years 1988 through 2019.^31^ We developed the ASL using data from the California Public Use Death Files from Years 1905 to 2020, and an extract of Armenian surnames from the Middle Eastern Surname List that was developed in 2007.^17^ The ASL contains 3428 unique surnames that can be linked with last names in research databases to identify Armenians.^17^ We also selected surnames from the CCR with birthplace in Armenia that were not in the ASL, and we linked this list of surnames to the CCR incidence research file extract. After manual review of both linkages, we combined the resulting matched records into one file. In the incidence file, we included all malignant cancer diagnoses among males and females using the *International Classification* of Diseases for Oncology, 3rd edition/World Health Organization (ICD‐O‐3/WHO) 2008 site codes.^32^, ^33^ We extracted Armenians from the NHW, non‐Hispanic Black, Hispanic, Asian/Pacific Islander, American Indian, Other/Unknown CCR race, and ethnicity categories to create a separate Armenian group. We included Armenian and NHW patients of all ages who were male and female due to the availability of population estimates by sex. We compared Armenians to the NHW group because 96.2% of patients we identified as Armenian were previously identified as NHW in the CCR and this historical categorization of Armenians as NHW has likely masked true cancer incidence among the Armenian population in California.

### 2.3 Statistical analysis

Demographic characteristics and cancer characteristics were stratified by sex and presented for the Armenian and NHW population. We calculated proportional incidence ratios (PIR), incidence rates (IR) and rate ratios (IRR) for the most frequent cancers among Armenian males and females compared with NHWs. Armenian population denominators were obtained from the American Community Survey (ACS) for IR calculations. The PIR is the observed number of Armenian cancer cases divided by the number of Armenian cancer cases expected if the Armenian population has the same proportion of cancer as the NHW population.^9^, ^34^, ^35^ Age‐adjusted PIRs were calculated for cancers diagnosed from 1988 to 2019 and from 2006 to 2015 to directly compare with IRRs available only for Years 2006–2015. Due to concerns raised previously regarding the accuracy of ACS data denominators for small populations, we calculated the PIR as the primary method in this study.^36^ The following PIR formula was used.

$$\mathrm{PIR}=\frac{\sum A_{i}}{\sum B_{i}\left(\frac{C_{i}}{D_{i}}\right)}$$

where $A_{i}$, number of Armenian site‐specific cases for age group ⅈ; $B_{i}$, total number of Armenian cases for age group ⅈ; $C_{i}$, number of NHW site‐specific cases for age group ⅈ; $D_{i}$, total number of NHW cases for age group ⅈ.

ACS Armenian population denominator estimates from 2006 to 2015 were used to obtain IRs per 100,000 people age‐adjusted to the US 2000 standard population in 18 age categories. We presented IRs and IRRs for the 10 most common cancers among Armenian males and females. We then compared the IRs of these top 10 cancers observed among Armenian males and females with the NHW racial and ethnic group as the reference population with IRRs and 95% confidence intervals during 2006–2015. SEER*Stat software (version 8.4.0) was used to retrieve age‐adjusted IRs, while SAS version 9.4 software (SAS institute Inc., Cary, NC, USA) was used to obtain descriptive demographic data, PIRs, and IRRs.

## 3 RESULTS

Relevant characteristics of Armenians diagnosed with cancer in California between Years 1988 and 2019 are presented in Table 1 and Table S2 is provided for comparison of characteristics with NHW patients. A total of 27,212 malignant cancer diagnoses among Armenians in California met inclusion criteria with nearly an equal frequency of cancer cases among males (50.5%) and females (49.5%). About 13% of diagnoses occurred among people less than 50 years old, 43% were between 50 and 69 years old, and 44% were aged 70 or greater. Most (76.3%) of the cancer cases were diagnosed in Los Angeles County, followed by Fresno (5.7%) and Orange (3.3%) counties. Most (55.2%) of the study population was foreign‐born, primarily in Armenia (20.1%), Iran (12.7%), Lebanon (4.3%), Syria (3.6%), and Russia (3.5%). Overall, 12.8% were United States‐born (US‐born) and 32.0% had unknown place of birth.

**TABLE 1:** Characteristics of Armenians diagnosed with cancer in California, California Cancer Registry, 1988–2019.

	Male	Female	Total
	(*n* = 13,754)	(*n* = 13,458)	(*N* = 27,212)
Characteristics	*n* (%)	*n* (%)	*N* (%)
Age at diagnosis			
0–9	69 (0.5)	51 (0.4)	120 (0.4)
10–19	66 (0.5)	65 (0.5)	131 (0.5)
20–29	157 (1.1)	173 (1.3)	330 (1.2)
30–39	312 (2.3)	574 (4.3)	886 (3.3)
40–49	717 (5.2)	1,380 (10.3)	2,097 (7.7)
50–59	2,048 (14.9)	2,474 (18.4)	4,522 (16.6)
60–69	4,002 (29.1)	3,197 (23.8)	7,199 (26.5)
70+	6,383 (46.4)	5,544 (41.2)	11,927 (43.8)
County			
Los Angeles	10,722 (78.0)	10,054 (74.7)	20,776 (76.3)
Fresno	724 (5.3)	840 (6.2)	1,564 (5.7)
Orange	441 (3.2)	465 (3.5)	906 (3.3)
San Diego	230 (1.7)	264 (2.0)	494 (1.8)
Sacramento	159 (1.2)	207 (1.5)	366 (1.3)
Santa Clara	162 (1.2)	202 (1.5)	364 (1.3)
San Francisco	145 (1.1)	179 (1.3)	324 (1.2)
Other	1,171 (8.5)	1,247 (9.3)	2,418 (8.9)
Country of birth			
Armenia^a^	2,775 (20.2)	2,687 (20.0)	5,462 (20.1)
United States	1,589 (11.6)	1,898 (14.1)	3,487 (12.8)
Iran	1,803 (13.1)	1,652 (12.3)	3,455 (12.7)
Lebanon	604 (4.4)	555 (4.1)	1,159 (4.3)
Syria	537 (3.9)	436 (3.2)	973 (3.6)
Russia	458 (3.3)	508 (3.8)	966 (3.5)
Turkey	463 (3.4)	346 (2.6)	809 (3.0)
Greece	244 (1.8)	211 (1.6)	455 (1.7)
Iraq	176 (1.3)	179 (1.3)	355 (1.3)
Egypt	116 (0.8)	115 (0.9)	231 (0.8)
Other	545 (4.0)	599 (4.5)	1,144 (4.2)
Unknown	4,444 (32.3)	4,272 (31.7)	8,716 (32.0)

^a^ Includes Asian Republics of the USSR.

Based on the Armenian population demographics in California as reported by the ACS, there were an estimated 241,323 Armenians in California from 2006 to 2010 and 259,430 from 2011 to 2015, representing a 7.5% population increase between the two time periods (Table S1). Based on the 2015 5‐year estimate, 61% of the population was <50 years old, 26% was 50–69 years old, and 13% was ≥70 years old. A majority (75.6%) of the population resided in Los Angeles County, followed by Fresno (3.9%) and Orange (3.5%) counties. A majority (60.7%) of the population was foreign‐born, primarily in Armenia (25.4%), Iran (18.2%), Lebanon (4.7%), Syria (2.7%), and Iraq (1.7%). More than a third (39.3%) of the population was born in the United States.

Among 13,754 Armenian males diagnosed with cancer between 1988 and 2019, the top 10 most frequent cancers in descending order were prostate (22.8%), lung (15.2%), colorectal (12.3%), bladder (11.1%), non‐Hodgkin lymphoma (NHL) (4.3%), stomach (3.9%), leukemia (3.8%), kidney (3.6%), pancreas (2.5%), and liver and intrahepatic bile duct (IBD) (1.9%). Among 13,458 Armenian females diagnosed with cancer, the top 10 most frequent cancers in descending order were breast (32.6%), colorectal (12.1%), lung (6.2%), uterine (6.1%), NHL (4.3%), thyroid (4.2%), ovary (3.7%), stomach (3.0%), pancreas (2.9%), and leukemia (2.8%) (Table 2). For cancers diagnosed from 1988 to 2019, Armenian males had a significantly higher proportion of stomach (PIR = 2.39, 95% CI = 2.19–2.60), bladder (PIR = 1.53, 95% CI = 1.45–1.61), colorectal (PIR = 1.29, 95% CI = 1.23–1.35), lung (PIR = 1.16, 95% CI = 1.11–1.21), leukemia (PIR = 1.16, 95% CI = 1.06–1.26), liver and IBD (PIR = 1.19, 95% CI = 1.05–1.34), and kidney (PIR = 1.11, 95% CI = 1.01–1.21) cancers, and a significantly lower proportion of prostate (PIR = 0.84, 95% CI = 0.81–0.87) cancer compared to NHW males. In addition, while thyroid cancer was not among the top 10 most frequent for Armenian males, we observed a higher proportion of thyroid (PIR = 1.45, 95% CI = 1.25–1.67) cancer among Armenian males compared with NHW males. From 1988 to 2019, Armenian females had a significantly higher proportion of stomach (PIR = 3.24, 95% CI = 2.93–3.57), thyroid (PIR = 1.47, 95% CI = 1.36–1.60), colorectal (PIR = 1.29, 95% CI = 1.23–1.35), pancreatic (PIR = 1.20, 95% CI = 1.09–1.33), leukemia (PIR = 1.20, 95% CI = 1.08–1.32), NHL (PIR = 1.15, 95% CI = 1.05–1.24), and ovarian (PIR = 1.14, 95% CI = 1.04–1.24) cancers, and a significantly lower proportion of lung (PIR = 0.49, 95% CI = 0.46–0.53) cancer compared to NHW females.

**TABLE 2:** Age‐adjusted proportional incidence ratios for the 10 most common cancers in Armenians compared to NHWs, California Cancer Registry, 1988–2019.

Cancer site	Observed	Expected	PIR	95% CI
Male				
Prostate	3,132	3,741.82	0.84^a^	(0.81–0.87)
Lung	2,088	1,799.24	1.16^a^	(1.11–1.21)
Colorectal	1,695	1,318.04	1.29^a^	(1.23–1.35)
Bladder	1,531	1,002.81	1.53^a^	(1.45–1.61)
NHL	587	622.39	0.94	(0.87–1.02)
Stomach	530	222.15	2.39^a^	(2.19–2.60)
Leukemia	519	448.59	1.16^a^	(1.06–1.26)
Kidney	501	452.73	1.11^a^	(1.01–1.21)
Pancreas	348	338.80	1.03	(0.92–1.14)
Liver and IBD	257	216.01	1.19^a^	(1.05–1.34)
Female				
Breast	4,380	4,349.37	1.01	(0.98–1.04)
Colorectal	1,634	1,269.37	1.29^a^	(1.23–1.35)
Lung	839	1,702.06	0.49^a^	(0.46–0.53)
Uterine	818	813.01	1.01	(0.94–1.08)
NHL	573	499.97	1.15^a^	(1.05–1.24)
Thyroid	564	382.58	1.47^a^	(1.36–1.60)
Ovary	498	437.15	1.14^a^	(1.04–1.24)
Stomach	400	123.56	3.24^a^	(2.93–3.57)
Pancreas	393	327.10	1.20^a^	(1.09–1.33)
Leukemia	385	321.59	1.20^a^	(1.08–1.32)

Abbreviations: IBD, intrahepatic bile duct, NHL, non‐Hodgkin lymphoma; PIR, proportional incidence ratio.

^a^ Statistically significant.

In Table 3, we show the IRR and PIR for the top 10 most common cancers among Armenian males and females diagnosed from 2006 to 2015. Our results show significantly higher IRRs of stomach (IRR = 1.78, 95% CI = 1.55–2.06), bladder (IRR = 1.13, 95% CI = 1.05–1.23), and colorectal (IRR = 1.12, 95% CI = 1.03–1.20) cancers, and significantly lower prostate (IRR = 0.64, 95% CI = 0.60–0.68), NHL (IRR = 0.72, 95% CI = 0.63–0.81), leukemia (IRR = 0.84, 95% CI = 0.73–0.96), kidney (IRR = 0.79, 95% CI = 0.69–0.89), liver and IBD (IRR = 0.72, 95% CI = 0.59–0.87), and pancreatic (IRR = 0.72, 95% CI = 0.62–0.86) cancers among Armenian males compared to NHW males. Armenian males had an insignificantly lower IRR for thyroid (IRR = 0.89, 95% CI = 0.72–1.12) cancer. PIRs for males followed a similar pattern as described in Table 2, except that PIRs for liver and IBD (PIR = 0.92, 95% CI = 0.75–1.11), leukemia (PIR = 1.10, 95% CI = 0.95–1.26), kidney (PIR = 1.05, 95% CI = 0.92–1.19), and thyroid (PIR = 1.19, 95% CI = 0.95–1.47) cancers were not statistically significant. Among Armenian females, we observed significantly higher IRRs of stomach cancer (IRR = 2.54, 95% CI = 2.15–2.99) and significantly lower IRRs of breast (IRR = 0.74, 95% CI = 0.70–0.77), lung (IRR = 0.36, 95% CI = 0.33–0.41), and uterine (IRR = 0.76, 95% CI = 0.68–0.85) cancers. PIRs for females followed a similar pattern as in Table 2.

**TABLE 3:** Age‐adjusted incidence rates and rate ratios and proportional incidence ratios of the top 10 cancers among Armenians compared with NHWs, California Cancer Registry, 2006–2015.

Cancer Site	Armenian		NHW		IRR (95% CI)	O	E	PIR (95% CI)
	Count	IR (95% CI)	Count	IR (95% CI)				
Male								
Prostate	1,169	80.0 (75.4–84.6)	131,406	125.3 (124.6–126.0)	0.64* (0.60–0.68)	1,169	1,409.16	0.83* (0.78–0.88)
Lung	832	57.7 (53.7–61.6)	59,198	59.9 (59.4–60.4)	0.96 (0.89–1.03)	832	643.42	1.29* (1.21–1.38)
Colorectal	711	50.3 (46.5–54.0)	44,391	45.1 (44.6–45.5)	1.12* (1.03–1.20)	711	479.65	1.48* (1.38–1.60)
Bladder	649	45.1 (41.7–48.8)	38,850	39.8 (39.4–40.2)	1.13* (1.05–1.23)	649	421.97	1.54* (1.42–1.66)
NHL	253	18.2 (15.9–20.2)	24,407	25.4 (25.1–25.7)	0.72* (0.63–0.81)	253	265.26	0.95 (0.84–1.08)
Stomach	198	13.9 (12.0–16.0)	7,692	7.8 (7.6–8.0)	1.78* (1.55–2.06)	198	83.19	2.38* (2.06–2.74)
Leukemia	211	15.7 (13.6–18.0)	17,606	18.8 (18.5–19.1)	0.84* (0.73–0.96)	211	192.27	1.10 (0.95–1.26)
Kidney	228	15.9 (13.9–18.2)	20,158	20.3 (20.0–20.6)	0.79* (0.69–0.89)	228	217.24	1.05 (0.92–1.19)
Pancreas	145	10.1 (8.5–11.9)	13,810	13.8 (13.6–14.1)	0.72* (0.62–0.86)	145	149.35	0.97 (0.82–1.14)
Liver and IBD	108	7.4 (6.1–9.0)	10,988	10.3 (10.1–10.5)	0.72* (0.59–0.87)	108	117.78	0.92 (0.75–1.11)
Female								
Breast	1,664	102.9 (97.8–108.0)	150,964	139.7 (138.9–140.4)	0.74* (0.70–0.77)	1,664	1,716.53	0.97 (0.92–1.02)
Colorectal	661	36.8 (33.9–39.7)	41,490	35.3 (34.9–35.6)	1.04 (0.96–1.13)	661	459.94	1.44* (1.33–1.55)
Lung	343	18.7 (16.7–20.7)	60,259	50.7 (50.3–51.2)	0.36* (0.33–0.41)	343	661.20	0.52* (0.47–0.58)
Uterine	330	19.4 (17.3–21.5)	29,217	25.6 (25.3–25.9)	0.76* (0.68–0.85)	330	327.29	1.01 (0.90–1.12)
NHL	257	14.9 (13.1–17.0)	18,780	16.6 (16.3–16.8)	0.90 (0.79–1.02)	257	210.09	1.22* (1.08–1.38)
Thyroid	254	17.3 (15.1–19.5)	16,646	19.1 (18.8–19.4)	0.91 (0.79–1.03)	254	204.43	1.24* (1.09–1.41)
Ovary	200	11.8 (10.2–13.7)	14,382	13.1 (12.9–13.4)	0.90 (0.78–1.04)	200	163.44	1.22* (1.06–1.41)
Stomach	152	8.7 (7.3–10.3)	4,041	3.4 (3.3–3.5)	2.54* (2.15–2.99)	152	44.63	3.41* (2.89–3.99)
Pancreas	168	9.2 (7.9–10.8)	12,790	10.5 (10.3–10.7)	0.88 (0.75–1.02)	168	139.64	1.20* (1.03–1.40)
Leukemia	164	9.7 (8.2–11.4)	12,108	11.2 (10.9–11.4)	0.87 (0.74–1.02)	164	136.33	1.20* (1.03–1.40)

Abbreviations: CI, confidence interval; E, expected; IBD, intrahepatic bile duct; IR, age‐adjusted incidence rate per 100,000 people; IRR, incidence rate ratio; NHL, non‐Hodgkin lymphoma; O, observed; PIR, age‐adjusted proportional incidence ratio.

* Statistically significant at *p* < 0.05.

## 4 DISCUSSION

To our knowledge, this is the first study showing cancer incidence specifically among the Armenian population in California. While other studies have previously included Armenians in a broad Middle‐Eastern^12^, ^23^ group in California, we were able to use our recently developed surname tool, the ASL,^17^ and birthplace data to identify Armenians diagnosed with cancer in California. Compared with individuals in the NHW category, where Armenians have most commonly been included, we found significant differences in proportional incidence and IRs of many cancers. Notable findings include higher proportional incidence of stomach, colorectal and thyroid cancer among Armenian males and females and tobacco‐related cancers among Armenian males. Our findings can guide healthcare professionals and researchers towards targeted cancer screening interventions and etiologic studies for these cancers in Armenians.

Among the 10 most frequent cancers, Armenian males had higher proportions of seven cancers and higher IRs of three cancers, and Armenian females had higher proportions of seven cancers and a higher IR of one cancer compared to NHWs. The correct interpretation of the two methods is crucial for a thorough understanding of our study results; PIR analyses show which cancers are proportionally higher among Armenians diagnosed with cancer in California compared to NHWs, while IRR analyses show risk of cancer incidence compared to NHWs. For instance, a higher PIR for a particular cancer among Armenians does not mean that the risk of the respective cancer is higher than NHWs, but rather that the proportion of that cancer is higher than it is among NHWs.^34^ The two methods convey different but equally important findings, as proportional analyses can guide use of cancer‐related healthcare resources within the Armenian population, and IR analyses can be used to discover and address risk factors for higher risk of certain cancer types among Armenians compared to NHWs. In the context of our study, we think our IR calculations should be interpreted with caution due to uncertainty regarding accuracy of ACS population estimates.^36^ While we encourage future studies to further refine Armenian population estimates, we believe the IR data can provide complementary information to our proportional incidence analyses.

Our observation of a substantially higher proportion of stomach cancer among Armenians suggests a need for further exploration into the etiology of this cancer among this population. Risk factors for stomach cancer include a diet high in salt, smoked, processed, grilled, or barbecued meat, and low in fruits, vegetables, and fiber.^37^, ^38^ Obesity, a known risk factor for stomach, colorectal, and multiple other cancers, is a known public health issue in Armenia, with over half the population being overweight (22%) or obese (29%).^39^ While the prevalence of obesity in the Armenian population of California or the United States is unknown, a previous study in a Los Angeles county hospital showed that Armenian patients had a higher proportion of hyperlipidemia compared to non‐Armenian patients that may be related to either a diet high in saturated fats, red meat, or alcohol, or a possible genetic predisposition for hyperlipidemia among Armenians.^40^ In addition to diet and obesity, tobacco and alcohol use, low physical activity, family history of stomach cancer, and environmental exposure to infections such as *Helicobacter pylori (H. pylori)* and hepatitis B are all known risk factors.^37^, ^38^ Some studies also suggest an association between *H. pylori* exposure and colorectal cancer, which was also proportionally higher among Armenians in our study.^41^ The highest stomach cancer IRs in the world have been observed in Eastern Asia followed by Central and Eastern Europe,^37^ and evidence suggests that first‐generation immigrants to the United States may have had higher exposure to these harmful environmental agents in comparison to second‐generation immigrants.^9^ Further, a study conducted at a hospital in Los Angeles county with a large patient population of Armenian immigrants found that Armenians made up 61% of all stomach cancer cases diagnosed at the hospital.^16^ Additionally, the study showed that Armenian immigrants from Armenia had lower rates of stomach cancer compared to immigrants from Middle‐Eastern and other countries, providing supporting evidence of environmental effects on this observed disparity, as opposed to a genetic predisposition for stomach cancer.^16^ Our findings of high stomach and colorectal cancer proportions suggest the need for further research considering environmental and generational factors, to better understand and address cancer risk factors among the Armenian population in California.

We also observed higher proportions of several tobacco‐related cancers particularly among Armenian males, including lung, bladder, leukemia, kidney, liver, and IBD.^42^ Tobacco‐use, mainly in the form of cigarettes, has been an ongoing public health issue among Armenian men.^43^ Armenian men are estimated to have the 12th highest smoking rate in the world, and 27% of the Armenian population, 51.8% of men and 1.6% of women, are current smokers.^43^, ^44^ While the percentage of women in Armenia who smoke is much lower than men, over half of women in Armenia are exposed to second‐hand smoke at home.^45^ In addition, among 30 low‐middle income countries studied, pregnant women in Armenia have the highest percentage (70%) of daily second‐hand smoke exposure.^45^, ^46^ As of 2022, lung cancer had the highest incidence in Armenia among all cancers studied.^47^ It was the most frequent cancer among men, and the fifth most frequent among women, accounting for 22.5% of new cases among men and 5% among women.^47^ In our study, lung cancer was the second most common among men and third most common among women. We also found that men had a significantly higher proportion, while women had a significantly lower proportion and IR of lung cancer compared to NHWs, and a possible explanation for this finding may be partly attributed to the higher percentage of smoking among NHW women in California (7%), compared to Armenian women in Armenia (1.6%).^44^ Further studies regarding tobacco‐use behaviors among Armenians in California are needed to understand whether the high smoking prevalence observed in Armenia remains a pattern post‐immigration. Our results, along with the high percentages of tobacco‐use, lung cancer, and multiple tobacco‐related cancers among men in Armenia,^48^ suggest that tobacco‐use behaviors among Armenian men in California warrant attention, including the implementation of culturally competent tobacco‐cessation programs.

Our findings of higher proportional incidence of thyroid cancer among Armenians are consistent with previous studies among the Middle‐Eastern group in California that included Armenians compared to NHWs.^9^, ^13^ Although the primary known risk factor for thyroid cancer is exposure to radiation, thyroid cancer is often preceded by benign thyroid disease.^49^ Iodine deficiency is a known risk factor for thyroid disease and may therefore be indirectly linked with the eventual development of thyroid cancer.^49^ Sources of radiation exposure over time in Armenia include the Chernobyl accident of 1986, the Armenian Nuclear Power Plant, tests of nuclear weapons in the 1950s and 1960s, mining, cosmic rays in mountainous regions, and natural radioactivity of urban soil.^50^ Research from the 1970s showed Armenia to be an iodine‐deficient country after discovering high prevalence of goiter among children and adults.^51^, ^52^ In 1995, a survey found that half of pregnant women and 40% of 6–12 year old children had goiter.^52^ In the 1990s, even after a major salt producer in Armenia iodized table salt, goiter prevalence remained high in the next 10 years.^51^ As such, in 2004, the Government of Armenia initiated a salt iodization strategy, requiring the fortification of food and salt with iodine.^51^ While this national strategy successfully reduced levels of iodine deficiency, recent studies have still observed possible high risk of thyroid disorders in Armenia.^53^ Our findings highlight the need to better understand the etiology of and risk factors for thyroid cancer among different generations of Armenians in California.

Our study has limitations. One limitation is that we used ACS data to calculate age‐adjusted IRs among Armenians and previous studies have shown inconsistent IRs that were calculated using ACS population estimates compared with SEER population estimates among small populations.^36^ Specifically, our population estimates would not capture Armenians not identified by the ancestry variable in ACS, potentially underestimating the numbers of Armenians in California. Given this limitation, we advocate for continued efforts in collecting accurate population estimates to further assess cancer incidence among Armenians to confirm our findings. Another limitation is the high percentage of missing country of birth in the CCR (32%), impacting our ability to compare birthplace between the CCR and ACS data. However, prior studies observed that those with missing country of birth in the CCR are more likely to be US‐born rather than foreign‐born,^54^, ^55^ suggesting that a significant proportion of the patients with unknown country of birth are actually US‐born. This is further supported by the only somewhat lower proportions of foreign‐born Armenians identified in the CCR (55.2%) than the ACS (60.7%–61.6%). While we used available father's last names and maiden names, in addition to last names, to identify Armenians with cancer, a common limitation we faced was the lack of mother's last name in the CCR, which may have prevented the identification of cancer patients who have Armenian mothers with an Armenian last name and fathers with non‐Armenian last names. In addition, Armenians with non‐Armenian last names, either due to marriage or other changes, may have not been identified particularly in cases of missing maiden names and father's last names. Additionally, the data we used to calculate IRs included Armenians from different generations, and different generations of Armenians likely have differing cancer incidence due to the effects of acculturation on cancer risk.^9^ While we did not have a “gold standard” list or contact people to confirm their ethnic origin for validation of the ASL, we evaluated the ASL previously, including using NamSor, an onomastic classification tool, and found that 81% of surnames had Armenia listed as the most or second‐most likely country of origin.^17^ Despite these limitations, a significant strength in our study was that this was the first research application of the newly developed ASL as a tool for identifying Armenians in large research databases that can be utilized by other cancer registries.

## 5 CONCLUSION

Our study findings highlight numerous cancer patterns among the Armenian population, including disproportionately higher stomach, colorectal, and thyroid cancers among men and women and tobacco‐related cancers among men. Our findings of significantly different IRs of several cancers among Armenians diagnosed between 2006 and 2015 have potential to guide healthcare professionals serving the Armenian community in California towards targeted cancer screening interventions. Most notably, the significantly higher IRRs and PIRs of stomach cancer observed among Armenian males and females provides further confirmation that public health interventions to understand stomach cancer etiology are necessary. In addition, our findings suggest the need for further research considering nativity and generational factors, to better understand and address cancer etiology among the Armenian population in California. We encourage researchers and healthcare professionals, particularly in areas of California with the largest Armenian populations, such as Los Angeles and Fresno counties, to use these data as a basis to examine cancers disproportionately impacting Armenian populations and for developing culturally competent interventions to address risk factors such as tobacco‐use, obesity, and exposure to harmful environmental agents.

## AUTHOR CONTRIBUTIONS

**Ani S. Movsisyan Vernon:** Conceptualization (equal); data curation (equal); formal analysis (lead); investigation (lead); methodology (lead); validation (lead); visualization (lead); writing – original draft (lead); writing – review and editing (lead). **Jeffrey S. Hoch:** Conceptualization (equal); investigation (equal); methodology (equal); project administration (equal); writing – original draft (equal); writing – review and editing (equal). **Laura Fejerman:** Conceptualization (equal); investigation (equal); methodology (equal); project administration (equal); writing – original draft (equal); writing – review and editing (equal). **Theresa Keegan:** Conceptualization (equal); investigation (equal); methodology (equal); project administration (lead); supervision (lead); writing – original draft (equal); writing – review and editing (equal).

## FUNDING INFORMATION

This research was not supported by grant funding.

## CONFLICT OF INTEREST STATEMENT

The authors made no disclosures.

## ETHICS STATEMENT

Approval for this study protocol including all methods was received from the Committee for the Protection of Human Subjects (CPHS), the Institutional Review Board (IRB) for the State of California Health and Human Services Agency. As the nature of the study is retrospective, waiver of informed consent was received from the Committee for the Protection of Human Subjects (CPHS), the Institutional Review Board (IRB) for the State of California Health and Human Services Agency. All methods were carried out in accordance with relevant guidelines and regulations.

## PRÉCIS

Our findings show higher stomach, colorectal and thyroid cancer incidence among males and females and tobacco‐related cancers among males. Further research is warranted to understand and address risk factors associated with specific cancers among Armenians in California.

## Supporting information


Table S1.

Table S2.

## Data Availability

The data that support the findings of this study are available from the California Department of Public Health and the California Cancer Registry. Access is granted through an application process by the management or data custodians (https://www.cdph.ca.gov/Programs/CHSI/Pages/Data‐Applications.aspx) and (https://www.ccrcal.org/retrieve‐data/).

## References

[1] Race/Ethnicity and the 2020 Census—Census 20/20. Accessed April 13, 2021. https://www.census2020now.org/faces‐blog/same‐sex‐households‐2020‐census‐r3976

[2] Bureau UC . State Population Totals and Components of Change: 2020‐2021. Accessed October 12, 2022. https://www.census.gov/data/tables/time‐series/demo/popest/2020s‐state‐total.html

[3] Fittante D . But Why Glendale? A history of Armenian immigration to Southern California. Calif Hist. 2017;94(3):1‐31. Accessed April 14, 2021. https://escholarship.org/uc/item/6v04w06x

[4] Yeretzian AS . A History of Armenian Immigration to America with Special Reference to Conditions in Los Angeles. University of Southern California; 1923.

[5] Klapheke AK , Carvajal‐Carmona LG , Cress RD . Racial/ethnic differences in survival among gastric cancer patients in California. Cancer Causes Control. 2019;30:687‐696. doi:10.1007/s10552-019-01184-0 31102083 PMC7172226

[6] Bergmans R , Soliman AS , Ruterbusch J , et al. Cancer incidence among Arab Americans in California, Detroit, and New Jersey SEER registries. Am J Public Health. 2014;104(6):83‐91. doi:10.2105/AJPH.2014.301954 24825237 PMC4062012

[7] Ellis L , Abrahao R , Mckinley M , et al. Colorectal cancer incidence trends by age, stage, and racial/ethnic group in California, 1990‐2014. Cancer Epidemiol Biomarkers Prev. 2018;27:1011‐1018. doi:10.1158/1055-9965.EPI-18-0030 30115679

[8] Ziadeh C , Ziogas A , Jiang L , Anton‐Culver H . Breast Cancer Characteristics in Middle Eastern Women Immigrants Compared with Non‐Hispanic White Women in California. JNCI Cancer Spectr. 2018;2(2):pky014. doi:10.1093/jncics/pky014 31360847 PMC6649784

[9] Ziadeh C , Ziogas A , Anton‐Culver H . Cancer risk in different generations of Middle Eastern immigrants to California, 1988‐2013. Int J Cancer. 2017;141:2260‐2269. doi:10.1002/ijc.30928 28801942 PMC7785134

[10] Aintablian H , Melkonian C , Galoustian N , et al. Why we should study the Armenian population: a goldmine of public health information. MOJ Public Health. 2018;7:11‐12. doi:10.15406/mojph.2018.07.00196

[11] Phelan J , Jones NA , Konya S , et al. 2015 National Content Test Race and Ethnicity Analysis Report. 2017.

[12] Nasseri K . Construction and validation of a list of common Middle Eastern surnames for epidemiological research. Cancer Detect Prev. 2007;31(5):424‐429. doi:10.1016/j.cdp.2007.10.006 18023539 PMC2212593

[13] Nasseri K . Thyroid cancer in the Middle Eastern population of California. Cancer Causes Control. 2008;19(10):1183‐1191. doi:10.1007/s10552-008-9185-y 18543070

[14] Our World in Data . Smoking. Accessed October 25, 2022. https://ourworldindata.org/smoking

[15] International Agency for Research on Cancer WHO . Cancer Today. 2020 Accessed February 7, 2023. https://gco.iarc.fr/today/online‐analysis‐map?v=2020&mode=population&mode_population=continents&population=900&populations=900&key=asr&sex=0&cancer=39&type=1&statistic=5&prevalence=0&population_group=0&ages_group%5B%5D=0&ages_group%5B%5D=17&nb_items=10&gr

[16] Glendale Adventist Medical Center . 2014 Cancer Services Annual Report. 2014.

[17] Movsisyan AS , Nasseri K , Keegan TH . Construction of the Armenian Surname List (ASL) for public health research. BMC Med Res Methodol. 2023;23:29. doi:10.1186/s12874-023-01848-1 36709252 PMC9883902

[18] B01001: Census Bureau Table. Accessed October 24, 2022. https://data.census.gov/cedsci/table?q=armenian&g=0100000US_0400000US06,06%240500000&tid=ACSDT5YSPT2015.B01001

[19] California Cancer Registry . About California Cancer Registry. Accessed December 9, 2019. https://www.ccrcal.org/learn‐about‐ccr/about‐cancer‐registries/

[20] California Cancer Registry, Chronic Disease Surveillance and Research Branch . Policies and Procedures for Access to and Disclosure of Confidential Data From the California Cancer Registry. 2014 http://www.ccrcal.org/Cancer_Reporting/Reporting_Legislation_and_Regulation.shtml

[21] Hofferkamp J . Standards for Cancer Registries Volume III. 2008 Accessed February 12, 2024. http://www.naaccr.org

[22] Tucker TC , Durbin EB , McDowell JK , Huang B . Unlocking the potential of population‐based cancer registries. Cancer. 2019;125(21):3729‐3737. doi:10.1002/cncr.32355 31381143 PMC6851856

[23] Nasseri K , Mills PK , Allan M . Cancer Incidence in the Middle Eastern Population of California, 1988–2004. Asian Pac J Cancer Prev. 2007;8:405‐411. 18159978 PMC2222861

[24] United states Census Bureau . American Community Survey: Data Suppression. 2016 Accessed February 12, 2024. https://www.census.gov/programs‐surveys/acs/technical‐documentation/data‐suppression.html

[25] Table 1. First, Second, and Total Responses to the Ancestry Question by Detailed Ancestry Code: 2000. Accessed January 19, 2023. www.census.gov/prod/cen2000/doc/sf3.pdf

[26] Frequently Asked Questions (FAQs) About Ancestry. Accessed February 9, 2023. https://www.census.gov/topics/population/ancestry/about/faq.html

[27] U.S Census Bureau . American Community Survey: Ancestry. Accessed February 9, 2023. https://www.census.gov/acs/www/about/why‐we‐ask‐each‐question/ancestry/

[28] U.S Census Bureau . American Community Survey and Puerto Rico Community Survey 2021 Code List.

[29] U.S. Census Populations With Bridged Race Categories. Accessed August 29, 2023. https://www.cdc.gov/nchs/nvss/bridged_race.html

[30] Race / Ethnic Population with Age and Sex Detail, 1970–1989 | Department of Finance. Accessed August 29, 2023. https://dof.ca.gov/forecasting/demographics/estimates/race‐ethnic‐population‐with‐age‐and‐sex‐detail‐1970‐1989/

[31] Download Match*Pro Software. Accessed April 13, 2021. https://surveillance.cancer.gov/matchpro/download

[32] Site Recode ICD‐O‐3/WHO . SEER Data Reporting Tools. 2008 Accessed November 8, 2020. https://seer.cancer.gov/siterecode/icdo3_dwhoheme/index.html

[33] Fritz A , Percy C , Jack A , et al. ICD‐O international classification of diseases for oncology first revision. 2013 Accessed September 1, 2023. www.who.int

[34] Boyle P , Parkin DM . Cancer registration: principles and methods. Statistical methods for registries. IARC Sci Publ. 1991;(95):126‐158. 1894318

[35] Schwartz KL , Kulwicki A , Weiss LK , et al. Cancer among Arab Americans in the metropolitan Detroit area. Ethn Dis. 2004;14:141‐146. Accessed July 9, 2020. http://digitalcommons.wayne.edu/fmhs_pubs/1/ 15002934

[36] Mantey J , Ruterbusch J , Meza R , Schwartz K . Cancer incidence trends using American community survey estimates are not consistent with SEER for small populations. Cancer Epidemiol. 2016;43:87‐91. doi:10.1016/j.canep.2016.06.014 27420630 PMC6849392

[37] Ilic M , Ilic I . Epidemiology of stomach cancer. World J Gastroenterol. 2022;28(12):1187‐1203. doi:10.3748/wjg.v28.i12.1187 35431510 PMC8968487

[38] Ma K , Baloch Z , He TTB , Xia X . Alcohol consumption and gastric cancer risk: a meta‐analysis. Med Sci Monit. 2017;23:238‐246. doi:10.12659/MSM.899423 28087989 PMC5256369

[39] Andreasyan D . The problem of being overweight among the Armenian population. Public Heal Panor. 2017;3(4):764‐771.

[40] Rostomian AH , Soverow J , Sanchez DR . Exploring Armenian ethnicity as an independent risk factor for cardiovascular disease: findings from a prospective cohort of patients in a county hospital. JRSM Cardiovasc Dis. 2020;9:1‐7. doi:10.1177/2048004020956853 PMC7498958 32983420

[41] Zuo Y , Jing Z , Bie M , Xu C , Hao X , Wang B . Association between helicobacter pylori infection and the risk of colorectal cancer a systematic review and meta‐analysis. Medicine (Baltimore). 2020;99(37):1‐8. doi:10.1097/MD.0000000000021832 PMC7489651 32925719

[42] Maguire FB , Movsisyan AS , Morris CR , Parikh‐Patel A , Keegan THM , Tong EK . Evaluation of cancer deaths attributable to tobacco in California, 2014‐2019. JAMA Netw Open. 2022;5(12):e2246651. doi:10.1001/jamanetworkopen.2022.46651 36515948 PMC9856507

[43] Ministry of Health Armenia, RTI International, United Nations Development Programme, Secretariat of the WHO Framework Convention on Tobacco Control, World Health Organization . The Case for Scaling up WHO FCTC Implementation Investment Case for Tobacco Control in Armenia. 2021 Accessed September 11, 2023. https://www.undp.org/sites/g/files/zskgke326/files/migration/am/ARMENIA‐FCTC‐INVESTMENT‐CASE‐ENGLISH.pdf

[44] Smoking Rates by Country. 2023 Accessed September 12, 2023. https://worldpopulationreview.com/country‐rankings/smoking‐rates‐by‐country

[45] Farrington J , Kontsevaya A , Fediaev D , et al. Prevention and Control of Noncommunicable Diseases in Armenia: The Case for Investment. 2019 Accessed September 13, 2023. http://www.euro.who.int/pubrequest

[46] Reece S , Morgan C , Parascandola M , Siddiqi K . Secondhand smoke exposure during pregnancy: a cross‐sectional analysis of data from demographic and health survey from 30 low‐income and middle‐income countries. Tob Control. 2019;28(4):420‐426. doi:10.1136/TOBACCOCONTROL-2018-054288 30026189 PMC10442074

[47] Zohrabyan D , Karapetyan N , Danielyan S , et al. Lung cancer in Armenia. J Thorac Oncol. 2023;18(4):402‐409. doi:10.1016/j.jtho.2022.12.015 36990573

[48] Arshakyan M , Rocchi M , Arshakyan G , Chiarantini L . Disparities in Cancer Incidence in Armenia, 2013‐2016: Setting priorities for preventive interventions. Int J Health Sci. 2018;6:158‐165. Accessed September 20, 2023. www.researchpublish.com

[49] Kitahara CM , Schneider AB . Epidemiology of thyroid cancer. Cancer Epidemiol Biomarkers Prev. 2022;31(7):1284‐1297. doi:10.1158/1055-9965.EPI-21-1440 35775227 PMC9473679

[50] Arakelyan VB , Khachatryan GE , Nalbandyan‐Schwarz AG , Mothersill CE , Seymour CB , Korogodina VL . Main radiation pathways in the landscape of Armenia. Int J Radiat Biol. 2023;99(8):1178‐1187. doi:10.1080/09553002.2023.2172623 36706217

[51] Hutchings N , Aghajanova E , Baghdasaryan S , et al. A stratified cross‐sectional cluster model survey of iodine nutrition in Armenia after a decade of universal salt iodization. Endocr Pract. 2019;25(10):987‐993. doi:10.4158/EP-2018-0634 31170368

[52] Rossi L , Branca F . Salt iodisation and public health campaigns to eradicate iodine deficiency disorders in Armenia. Public Health Nutr. 2003;6(5):463‐469. doi:10.1079/PHN2003461 12943562

[53] Ghukasyan NN . Management of pregnancy and the possibility of normal delivery in pregnant women with thyroid cancer in Armenia. World J Adv Res Rev. 2022;16(3):634‐637. doi:10.30574/wjarr.2022.16.3.1365

[54] Gomez SL , Glaser SL , Kelsey JL , Lee MM . Bias in completeness of birthplace data for Asian groups in a population‐based cancer registry (United States). Cancer Causes Control. 2004;15(3):243‐253. doi:10.1023/B:CACO.0000024244.91775.64/METRICS 15090719

[55] Gomez SL , Glaser SL . Quality of cancer registry birthplace data for Hispanics living in the United States. Cancer Causes Control. 2005;16(6):713‐723. doi:10.1007/S10552-005-0694-7/METRICS 16049810

