# Supplementary material for: Cancer incidence among Armenians in California
Source: Cancer Med. 2024 Mar 16;13(5):e7100. doi: 10.1002/cam4.7100 (PMC10943375; doi:10.1002/cam4.7100)
Supplement: Supplementary file 1 — Table S1. [file CAM4-13-e7100-s002.docx]

#### Supplemental Table 1. Armenian Population demographics in California, American Community Survey, 2006-2015

|  | 2006-2010 | | 2011-2015 | |
| --- | --- | --- | --- | --- |
| Characteristics | N | % | N | % |
| **Population** |  |  |  |  |
| California population | 36,637,290 |  | 38,421,464 |  |
| Armenian population | 241,323 | (0.7) | 259,430 | (0.7) |
| **Sex** |  |  |  |  |
| Male | 117,867 | (48.8) | 125,075 | (48.2) |
| Female | 123,456 | (51.2) | 134,355 | (51.8) |
| **Age, y** |  |  |  |  |
| 0 to 9 | 24,222 | (10.0) | 25,183 | (9.7) |
| 10 to 19 | 30,333 | (12.6) | 29,099 | (11.2) |
| 20 to 29 | 36,357 | (15.1) | 38,276 | (14.8) |
| 30 to 39 | 30,122 | (12.5) | 34,539 | (13.3) |
| 40 to 49 | 35,819 | (14.8) | 32,731 | (12.6) |
| 50 to 59 | 34,985 | (14.5) | 38,764 | (14.9) |
| 60 to 69 | 21,885 | (9.1) | 27,920 | (10.8) |
| 70+ | 27,600 | (11.4) | 32,918 | (12.7) |
| **County** |  |  |  |  |
| Los Angeles | 179,279 | (74.3) | 196,075 | (75.6) |
| Fresno | 10,602 | (4.4) | 10,052 | (3.9) |
| Orange | 10,226 | (4.2) | 9,136 | (3.5) |
| San Diego | 5,503 | (2.3) | 5,292 | (2.0) |
| Santa Clara | 3,349 | (1.4) | 3,098 | (1.2) |
| Sacramento | 3,318 | (1.4) | 5,440 | (2.1) |
| San Mateo | 2,791 | (1.2) | 2,379 | (0.9) |
| Other | 18,856 | (7.8) | 21,605 | (8.3) |
| Unknown | 7,399 | (3.1) | 6,353 | (2.4) |
| **Nativity** |  |  |  |  |
| Foreign-Born | 148,638 | (61.6) | 157,566 | (60.7) |
| United States-Born | 92,685 | (38.4) | 101,864 | (39.3) |
| **Country of Birth** | | | | |
| United States | 92,685 | (38.4) | 101,864 | (39.3) |
| Armenia | 61,300 | (25.4) | 65,776 | (25.4) |
| Iran | 39,817 | (16.5) | 47,197 | (18.2) |
| Lebanon | 12,606 | (5.2) | 12,267 | (4.7) |
| Syria | 7,733 | (3.2) | 6,947 | (2.7) |
| Iraq | 4,484 | (1.9) | 4,369 | (1.7) |
| Turkey | 3,156 | (1.3) | 2,749 | (1.1) |
| Russia | 2,910 | (1.2) | 3,023 | (1.2) |
| Other | 16,632 | (6.9) | 15,238 | (5.9) |

**Source: American Community Survey 2010 and 2015 5-Year Selected Population Profile.**^49^
