# Supplementary material for: Cancer incidence among Armenians in California
Source: Cancer Med. 2024 Mar 16;13(5):e7100. doi: 10.1002/cam4.7100 (PMC10943375; doi:10.1002/cam4.7100)
Supplement: Supplementary file 2 — Table S2. [file CAM4-13-e7100-s001.docx]

Supplemental Table 2. Characteristics of Armenian and NHW patients diagnosed with cancer in California, California Cancer Registry, 1988-2019

|  | Armenian |  | NHW |  |
| --- | --- | --- | --- | --- |
|  | (n=27,212) |  | (n=3,094,510) |  |
| Characteristics | n | % | n | % |
| **Year of Diagnosis** |  |  |  |  |
| 1988-1998 | 5,572 | (20.5) | 1,025,176 | (33.1) |
| 1999-2009 | 9,658 | (35.5) | 1,081,555 | (35.0) |
| 2010-2019 | 11,982 | (44.0) | 987,779 | (31.9) |
| **Age at Diagnosis** |  |  |  |  |
| 0 to 9 | 120 | (0.4) | 9,705 | (0.3) |
| 10 to 19 | 131 | (0.5) | 10,663 | (0.3) |
| 20 to 29 | 330 | (1.2) | 34,552 | (1.1) |
| 30 to 39 | 886 | (3.3) | 92,192 | (3.0) |
| 40 to 49 | 2,097 | (7.7) | 223,596 | (7.2) |
| 50 to 59 | 4,522 | (16.6) | 484,491 | (15.7) |
| 60 to 69 | 7,199 | (26.5) | 801,715 | (25.9) |
| 70+ | 11,927 | (43.8) | 1,437,596 | (46.5) |
| **Sex** |  |  |  |  |
| Male | 13,754 | (50.5) | 1,600,579 | (51.7) |
| Female | 13,458 | (49.5) | 1,493,931 | (48.3) |
| **Cancer Type** |  |  |  |  |
| Breast | 4,422 | (16.3) | 478,260 | (15.5) |
| Colorectal | 3,329 | (12.2) | 300,226 | (9.7) |
| Prostate | 3,132 | (11.5) | 434,593 | (14.0) |
| Lung | 2,927 | (10.8) | 405,193 | (13.1) |
| Bladder | 1,893 | (7.0) | 152,827 | (4.9) |
| NHL | 1,160 | (4.3) | 129,014 | (4.2) |
| Stomach | 930 | (3.4) | 40,212 | (1.3) |
| Leukemia | 904 | (3.3) | 86,913 | (2.8) |
| Uterine | 818 | (3.0) | 89,563 | (2.9) |
| Kidney | 764 | (2.8) | 81,330 | (2.6) |
| Other | 6,933 | (25.5) | 896,379 | (29.0) |
| **County** |  |  |  |  |
| Los Angeles | 20,776 | (76.3) | 607,854 | (19.6) |
| Fresno | 1,564 | (5.7) | 60,976 | (2.0) |
| Orange | 906 | (3.3) | 274,888 | (8.9) |
| San Diego | 494 | (1.8) | 295,728 | (9.6) |
| Sacramento | 366 | (1.3) | 132,726 | (4.3) |
| Santa Clara | 364 | (1.3) | 133,973 | (4.3) |
| San Francisco | 324 | (1.2) | 68,389 | (2.2) |
| Other | 2,418 | (8.9) | 1,519,976 | (49.1) |
| **Nativity** |  |  |  |  |
| United States-Born | 3,487 | (12.8) | 1,575,466 | (50.9) |
| Foreign-born | 15,009 | (55.2) | 177,886 | (5.7) |
| Unknown | 8,716 | (32.0) | 1,341,158 | (43.3) |

NHL: non-Hodgkin lymphoma

IBD: intrahepatic bile duct
